# Effect of pre-analytical treatments on bovine milk acute phase proteins

**DOI:** 10.1186/s12917-016-0769-6

**Published:** 2016-07-25

**Authors:** Funmilola C. Thomas, Andre M. Santana, Mary Waterston, Hayley Haining, Peter David Eckersall

**Affiliations:** 1 Institute of Biodiversity Animal Health and Comparative Medicine, University of Glasgow, Bearsden Road, Glasgow, UK; 2 Department of Veterinary Clinics and Surgery, FCAV, UNESP, Sao Paulo, Brazil; 3 Institute of Infection, Immunity and Inflammation, GBRC University Place, University of Glasgow, Glasgow, UK; 4 Veterinary Diagnostic Services, School of Veterinary Medicine, University of Glasgow, Bearsden Road, Glasgow, UK; 5 Present Address: Department of Veterinary Physiology and Pharmacology, College of Veterinary Medicine, Federal University of Agriculture, Abeokuta, Nigeria

**Keywords:** C-reactive protein, Haptoglobin, Thermal stability, Mammary associated serum amyloid A3, Milk preservatives

## Abstract

**Background:**

Samples for diagnostic procedures often require some form of pre-analytical preparation for preservation or safe handling during transportation prior to analysis in the laboratory. This is particularly important for milk samples which frequently need preservatives to retain milk composition as close to that found in freshly collected samples as possible.

**Methods:**

Milk samples were treated by heating at 56 °C for 30 min or preserved by addition of either potassium dichromate or bronopol respectively. Haptoglobin (Hp), mammary associated serum amyloid A3 (M-SAA3) and C-reactive protein (CRP) were measured in the various treatment groups and in control samples which were not treated, using enzyme linked immunoassays. The concentrations of each APP were compared between treated and non-treated groups using the Wilcoxon signed ranks tests.

**Results:**

Heat treatment of samples was found to have a significant lowering effect on milk M-SAA3 and CRP but not Hp. The use of potassium dichromate and bronopol as preservatives in milk had no significant effects on milk Hp and M-SAA3 concentration but lowered milk CRP values compared to controls.

**Conclusions:**

The observed effects of heating and preservative use on milk APP should be taken into consideration when assaying samples which have undergone heat treatment as a result of international transfer regulations involving biological samples or samples needing chemical preservation prior to transport to laboratory.

## Background

Acute phase proteins are continuing to gain importance particularly as diagnostic tools in veterinary medicine [1]. With the discovery of the major APP in bovine milk and its correlation with mastitis [2], there has been a growing interest of their exploitation as biomarkers of bovine mastitis which can be adapted to a rapid, on farm measuring format. Given that bovine mastitis is one of the most prevalent and costly conditions affecting dairy cows which greatly impacts on the economics, welfare and public health aspects of dairy farming, the substantiation of APP for mastitis diagnosis is on-going and methods to apply their measurement to a rapid format has been explored [3, 4].

Acute phase proteins are proteins that are synthesized in the liver and other organs in response to inflammatory stimuli and released into body fluids such as serum, milk [1], ovarian fluid [5] among others. These samples are therefore collected and assayed for APP in order to make clinical diagnosis of inflammatory and other disease conditions.

Samples for diagnostic procedures often require some form of preparation, preservation or safe handling for transportation prior to analysis in the laboratory. This is especially true for milk samples which frequently need the use of preservatives to retain milk composition as close to that found in freshly collected samples as possible. Chemical preservation is used to avoid cellular degradation prior to somatic cell counts (SCC) or progesterone analysis (pregnancy test). Storage at freezing temperatures of between −20 °C and −80 °C is also a common way of preserving milk samples.

In addition, as a requirement for the safe transfer of biological samples across international borders, some national regulations demand heat treatment of liquid samples in order to inactivate harmful pathogens in the samples have been put in place (for example; Council directive 82/894/EEC of 21 December 1982, under the European Communities Act 1972).

However, high temperature is known to denature proteins and to influence the concentration and immunologic activities of some serum proteins [6]. Heat treatment has also been shown to result in irreversible changes in the structure of milk proteins [7]. In addition, heating of milk has been shown to affect various characteristics of milk ranging from allergenicity of milk proteins [8, 9] to the concentration of water soluble vitamins, as well as concentration and activity of some milk proteins [10–12]. Furthermore, it was observed that heating colostrum to 60 °C for 60 min resulted in inactivation of pathogens and loss (although minimal) in colostral IgG [11]. A number of other studies have also focused on the effect of heat on residues of drugs (quninolones, aminoglycosides) in milk [13, 14].

In the handling of milk samples for analysis, the use of preservatives has become a requirement for some tests due to distances of laboratories from the farms where samples are obtained. Potassium dichromate (K_2_Cr_2_O_7_) as in Lactab Marks III® is a corrosive and toxic biocide which is often used in the preservation of milk samples in order to maintain milk composition from time of collection to analysis. It is known to be effective as a milk preservative [15], and is used to maintain progesterone levels in milk for pregnancy diagnosis. Another broad spectrum milk preservative, bronopol (C_3_H_6_BrNO_4_; 2-bromo-2-nitro-1, 3 propanediol), which unlike potassium dichromate is reported to have no toxic effects, is often used particularly for preserving milk somatic cells prior to analysis through its action as an antimicrobial agent [16, 17]. Other preservatives that have been used in milk samples include Mercury chloride (HgCl_2_), sodium azide [18], Azidiol [19] and formalin [20].

Although the efficacy of these milk preservatives in maintaining milks’ natural composition including protein, fat and SCC has been proven [21–23], there has also been early reports [24–26] of alterations in analyte concentrations following the use of these preservatives. For example potassium dichromate was shown to lead to a decrease in protein concentration within 24 h after milk collection [27]. In addition, bronopol based preservatives were shown to alter the mid infrared measurements of protein contents of milk samples [15]. In another study however, the use of the preservatives potassium dichromate, bronopol and sodium azide did not significantly change fats, proteins, lactose and milk solids content of milk when samples were analyzed within 7 days of preservative use, but were reported to lead to a decline in these components when measured in samples stored for over 7 days after preservation [17]. Use of bronopol has also been reported to yield higher values of % proteins and fat compared to values measured in aliquots of the samples following the use of potassium dichromate [25]. Differences in effects on milk microflora between use of different preservatives have also been reported [28]. Other parameters that have been reported to be affected by chemical preservative use include the electrical conductivity (EC) of milk by potassium dichromate [29].

From these reports, it can be inferred that there is a possibility that the use of chemical preservatives may affect analytes in milk. Indeed it has been recommended that where the preservative used in milk interferes with the analyte being tested; a suitable correction factor should be used [30].

It is not known if the use of chemical preservatives in milk meant for analysis or heat treatment of milk samples in order to inactivate pathogens before international transit, has any effect on the concentration of milk APP. It is important to be aware of such effects if they exist, if such treated milk samples are to be used for analysis of APP.

This study was carried out to evaluate the effects of heat treatment and chemical preservative use on milk APP concentration. This consisted of the assessment of milk Hp, M-SAA3 and CRP following the use of the milk preservatives; potassium dichromate (Lactab Marks III®) and bronopol (broad spectrum Microtab® II) for milk preservation after 24 h and heating samples to pathogen-inactivation temperature (56 °C) for 30 min.

## Methods

### Samples

Milk samples were residual samples from previously described investigations [31] and also the residue of milk from commercial dairy farms in the West of Scotland submitted to Veterinary Diagnostic Services, University of Glasgow, for evaluation for mastitis. The samples covered a wide concentration range of APP; encompassing low values in non-mastitic milk and high values as expected in mastitic milk samples.

After mastitis diagnosis the residual of each sample was made into aliquots, stored at −20 °C before being subjected to various pre-analytical treatments before APP determination.

### Sample treatments

Aliquots (~500 μl) were treated in one of the following ways described below, while control sample aliquots were not treated.

Heat treatment was carried out by heating milk samples at 56 °C for 30 min in a water bath after which they were analyzed for Hp (*n* = 59), M-SAA3 (*n* = 44) and CRP (*n* = 53) alongside controls (unheated).

To evaluate the effect of preservatives, another set of milk aliquots had 1 mg of potassium dichromate (Lactabs Mark III®, Thompson and Capper Ltd, Cheshire, UK) per 10 ml of milk sample added and allowed to gently mix and act for 24 h at 4–8 °C on a shaker. Subsequently samples were analyzed for Hp (*n* = 35), M-SAA3 (*n* = 18) and CRP (*n* = 20). A different set of milk aliquots had 8 mg (1 tablet) of bronopol (Broad spectrum Microtabs®, Advanced instruments, *Inc.*, Massachusetts, USA) added to 40 ml of milk sample and gently mixed for 24 h at 4–8 °C. Hp (*n* = 54), M-SAA3 (*n* = 21) and CRP (*n* = 21) were also measured in these samples. Control samples (without any form of treatment) were also held at 4–8 °C for 24 h before analysis.

### Acute phase protein assays

#### Haptoglobin

Samples were assayed for Hp using an in-house ELISA developed for measuring milk Hp as described in Thomas et al.[31]. Dilutions were varied (ranging from 1:200 to 1:3200) from sample to sample depending on its Hp concentration, in order for the optical density value to fall within the interpolating range of the standard curve.

#### Mammary associated serum amyloid A

Tridelta Development Ltd supplied the Phase™ range SAA ELISA kit. Assay was performed as described in Thomas et al. [31]. Sample dilutions were also modified to accommodate samples with very high M-SAA3 concentration samples (range of dilutions were from 1:125 to 1: 1000).

#### C-reactive protein

Cow C-reactive protein (CRP) ELISA kits for assay of bovine milk CRP were supplied by the Life Diagnostics *Inc.* (West Chester, USA). The assay was performed as described in Thomas et al.[31]. Sample dilutions ranged from 1: 5 to 1:50.

### Statistical analyses

Tests for normality of each APP data were carried out using the Kolmogorov–Smirnov and Shapiro–Wilk test along with normal probability plots and quantile-quantile (Q-Q) plots.

To assess the effects on each milk APP of heat treatment and use of preservatives: potassium dichromate and bronopol, the Wilcoxon signed ranks test (non-parametric) was used to compare treated and untreated groups. The Spearman’s rho correlation test was also carried out to assess the correlation between each APP in the different treatment groups. A P value of <0.05 was considered significant. All statistical analyses were carried out using the IBM statistical package for social sciences (SPSS) software; version 21 (IBM Corporation USA, 2012).

## Results

Table 1 shows the statistical observations from the comparison of three acute phase proteins values between various treated and untreated milk samples.

**Table 1:** Effect of heat treatment and preservative use on Hp, M-SAA3 and CRP values in bovine milk

Protein	Treatment	Significant difference P	Regression equation in the form of y = c + mx^a^	Correlation coefficient R^2/^P
Hp	Heat *n* = 59	0.066	Heated Hp = 13.05 + 0.76 x unheated Hp	0.85/*P* < 0.001
SAA	Heat *n* = 44	0.000	Heated SAA = 38.6 + 0.28 unheated SAA	0.925/ *P* < 0.001
CRP	Heat *n* = 53	0.007	Heated CRP = −57.69 + 0.61 unheated CRP	0.932/*P* < 0.001
Hp	Potassium dichromate *n* = 35	0.080	Treated Hp = −270 + 1.04 non treated Hp	0.982/*P* < 0.001
SAA	Potassium dichromate *n* = 18	0.363	Treated SAA = −0.30 + 0.99 non treated SAA	0.642/*P* < 0.05
CRP	Potassium dichromate *n* = 20	0.003	Treated CRP = 5.41 + 1.47 non treated	0.913/*P* < 0.001
Hp	Bronopol *n* = 54	0.155	Treated Hp = 52.4 + 0.95 non treated Hp	0.882/ *P* < 0.001
SAA	Bronopol *n* = 21	0.088	Treated SAA = 1.10 + 0.0005 non treated	0.262/*P* > 0.05
CRP	Bronopol *n* = 21	0.028	Treated CRP = 2.15 + 0.96 non treated	0.765/*P* < 0.001

^a^y = treated group, x = non treated group, c = y value when x = 0, m = gradient

### Haptoglobin

The intra-assay and inter-assay precision of the Hp ELISA were 6 % and 27 % correspondingly while the limit of detection (LOD) of the assay was 0.4 μg/ml as reported in Thomas et al.[31].

**Fig. 1:**
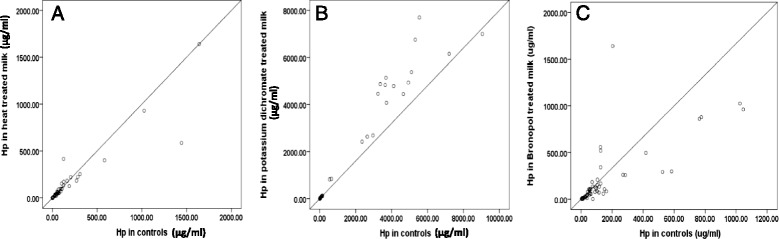
**a** Regression plot of Hp concentrations in heated treated versus unheated milk samples (*n* = 59); (**b**) Regression plot of Hp concentration in potassium dichromate treated versus untreated milk samples (*n* = 35); (**c**): Regression plot of Hp concentrations in bronopol treated versus untreated milk samples (*n* = 54)

No significant difference was observed between the milk Hp concentration of heated and unheated samples (*P* = 0.06) and a significant correlation between heated and unheated samples’ Hp was observed. The use of potassium dichromate and bronopol as milk preservatives for a duration of 24 h also did not result in a significant difference in milk Hp from controls (Table 1). Regression curves of concentrations of Hp in treated versus untreated milk samples are shown Fig. 1a-c. Table 2 shows the means and standard deviations of the concentrations in control and treated samples of Hp MSAA3 and CRP in the different treatment protocols.

**Fig. 2:**
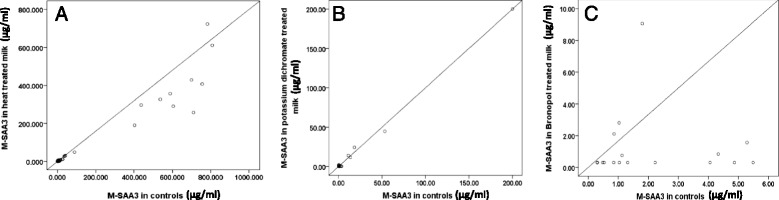
**a** Regression plot of MSAA3 in heated versus unheated milk samples (*n* = 44); (**b**) Regression plot of MSAA3 concentrations in potassium dichromate treated versus untreated milk samples (*n* = 18); (**c**) Regression plot of MSAA3 concentrations in bronopol treated versus untreated milk samples (*n* = 21)

**Fig. 3:**
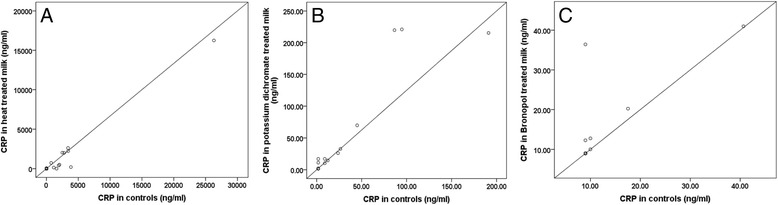
**a** : Regression plot of CRP concentrations in heated versus unheated milk samples (*n* = 53); (**b**) Regression plot of CRP in potassium dichromate treated versus untreated milk samples (*n* = 20); (**c**) Regression curve of CRP in bronopol treated versus untreated milk samples (*n* = 21)

**Table 2:** Means and standard deviations of APP concentrations in controls versus treatments with heat, potassium dichromate and bronopol

Protein	Treatment	Total (n)	Mean ± SD of controls	Mean ± SD of treated
Hp (μg/ml)	Heat	59	130.50 ± 311.86	112.88 ± 257.95
Hp (μg/ml)	Potassium dichromate	35	1533.94 ± 2144.03	1791.14 ± 2483.71
Hp (μg/ml)	Bronopol	54	160.29 ± 245.48	204. ± 321.11
MSAA3 (μg/ml)	Heat	44	153.72 ± 270.84	94.39 ± 178.48
MSAA3 (μg/ml)	Potassium dichromate	18	17.16 ± 47.40	16.76 ± 47.20
MSAA3 (μg/ml)	Bronopol	21	1.94 ± 1.80	1.10 ± 2.05
CRP (ng/ml)	Heat	53	939.57 ± 3.69	514.86 ± 2.28
CRP (ng/ml)	Potassium dichromate	20	25.88 ± 47.68	43.56 ± 77.20
CRP (ng/ml)	Bronopol	21	11.01 ± 7.04	12.74 ± 9.03

### Mammary associated serum amyloid A

Intra-assay and inter-assay CV of the MSAA3 ELISA was 7 % and 33 % respectively and a LOD of 0.6 μg/ml was established for the assay [31]. The M-SAA3 concentrations in heated samples were significantly different (lower) than those recorded from the unheated samples. However use of either potassium dichromate or bronopol preservatives did not indicate a significant difference in the values of M-SAA3 in milk from control values (Table 1). Regression curves of concentrations of M-SAA3 in treated versus untreated milk samples are shown Fig. 1b.

### C-reactive protein

Intra-assay and inter-assay CV of the CRP assay was 4 % and 7 % respectively and the LOD was 1.8 ng/ml [31]. Heating milk samples was also observed to have a significant lowering effect on milk CRP values (*P* = 0.007). A significant correlation was observed between CRP values in heated and unheated milk samples (*r* = 0.97). The use of potassium dichromate and bronopol in milk was shown to produce a significant increase in milk CRP values compared to controls (*P* = 0.003, and 0.028 respectively) (Table 1). Regression curves of concentrations of CRP in treated versus untreated milk samples are shown Fig. 1c.

## Discussion

In this study, three pre-analytical treatments regimes were carried out on milk samples with the aim of comparing the APP values in treated and untreated samples.

Studies have shown that some protocols of heat treatment could have significant reduction effects on milk (colostral) proteins especially IgG [32, 33] and on lactoferrin [34]. The observed lowering of M-SAA3 and CRP concentrations in heated samples and changes in CRP values of chemically preserved milk in the present study, lends further credence to these observations. It is generally known that high temperatures can result in the irreversible change in milk proteins structure [7], moreover it has been shown that aggregation of whey proteins can result upon heating of milk [35], this may explain some of the changes observed in the concentration of protein analytes in milk (which are mainly contained in the whey fraction).

It has been shown that high temperatures, can result in dissociation of CRP pentameres into its monomeric forms in blood [36], and although this may not affect the total concentration of serum CRP, it signals possible protein structural changes that are possible upon heating. There has been no report of the effect of heating on the structure of bovine milk CRP or M-SAA3. It can be inferred that a denaturation effect of heat on the protein structure or moiety of these compounds as reported by Hausen et al. [6] causes a loss of functional ability of these proteins to bind antibodies.

This finding should therefore be taken into consideration when assaying for M-SAA3 and CRP in samples which have undergone heating as a result of international transfer regulations involving heat treatment of biological samples. In such studies, control samples should be treated in the same manner as experimental samples or a correction factor based on the reduction in concentration should be applied. Structural changes in Haptoglobin have been associated with increased body temeprature (heat shock conditions) [37]. However, no significant change in milk Hp concentration due to heating was observed in this study. Studies have shown good correlation between milk Hp and SCC [31, 38], and in the study of Hachana et al. [39], heating milk samples to 60 °C prior to analysis did not alter the Fossomatic SCC, although the duration for which samples were held at that temperature before analysis was not ascertained.

Only CRP concentrations were affected by the use of chemical preservatives in milk, with the concentration showing a significant increase following treatment with both potassium dichromate and Bronopol but the reason for this is not clear. Therefore samples being collected for SCC or for progesterone analysis can also be used for determination of Hp and M-SAA3 concentrations but should be cautiously used for CRP determination.

In this study, a minimum time of 24 h was used to assess the possible preservative effects of potassium dichromate and Bronopol and it is possible that samples preserved for longer (beyond 24 h) may develop changes that could significantly alter milk concentration of Hp and M-SAA3 as observed for some other proteins by Chalermsan et all [17]. The milk used in this study were residual samples collected for either clinical investigation or prior experimental study and selected to give a range of APP concentrations. The effect of chemical preservatives was assessed after 24 h mixing and incubation at 4–8 °C in order replicate to some degree the likely treatment of samples between collection addition of preservative and analysis. While it would be ideal to assess the effect of heat and chemical preservation on fresh samples treated immediately, this was beyond the scope of the present investigation. In future investigation of APP in milk, where preservatives are being used to allow SCC or progesterone analysis, it would be of value to assess the effects on APP under a variety of conditions. Nevertheless, it is recommended that samples needing to be transported over long distances without any means of cold-chain storage, can be preserved using either potassium dichromate (Lactabs Mark III) or Bronopol (Microtabs), but should be analyzed as soon as possible and a correction factor should be applied in cases of CRP assay. It would be interesting to examine the effect of prolonged storage of milk with preservatives on milk APP.

## Conclusions

Heating milk samples and preservative use in milk can affect the measurement of APP concentration. However, if this is recognized and taken into consideration when assaying milk samples which have undergone heat treatment, as maybe required to conform with international transport regulations, or when assaying samples needing chemical preservation, then APP analysis will give valid results especially when control samples are treated with identical procedures.

## Abbreviations

APP, acute phase proteins; APR, acute phase response; CRP, C-reactive protein; CV, coefficient of variation; ELISA, enzyme linked immunosorbent assay; Hp, haptoglobin; IMI, intramammary infection/inflammation; LOD, limit of detection; M-SAA3, mammary associated serum amyloid A3; SAA, serum amyloid A; SCC, somatic cell counts; SPSS, statistical package for social sciences; x, times
